# The First Reported Case of Hafnia alvei Granulomatous Mastitis in Humans

**DOI:** 10.7759/cureus.70989

**Published:** 2024-10-07

**Authors:** Suhair Al Saad, Hamdi Al Shenawi, Eman Farid, Fatima Al Shenawi, Huda Shaker, Noor Al Shenawi

**Affiliations:** 1 Surgery, Arabian Gulf University, Manama, BHR; 2 Pathology/Immunology, Arabian Gulf University, Manama, BHR

**Keywords:** animal origin, biopsy, culture, granulomatous, hafnia alvei, imaging, mastitis

## Abstract

Granulomatous mastitis (GM) is an uncommon, chronic inflammatory breast disease, mostly idiopathic. Occasionally, the causative organism is isolated. It is characterized histologically by polymorph nuclear neutrophils' predominance and the absence of caseous necrosis. Idiopathic GM could be associated with other general conditions like autoimmune disorders, diabetes mellitus, and sarcoidosis. We report a case of a 41-year-old woman who presented with a left breast abscess of two weeks duration and a three-year history of intermittent attacks of painful breast swelling, which required multiple and extended empirical courses of antibiotics. Diagnosis as GM caused by Hafnia alvei bacteria was only confirmed by biopsy of the inflammatory mass and culture of the pus. The course of treatment was over three years, with three relapses.

## Introduction

Granulomatous mastitis (GM) is a rare, chronic inflammatory breast disease with an idiopathic etiology. It is diagnosed by exclusion [1-4]. The usual presentation of GM is a firm, unilateral, and distinct breast swelling accompanying an abscess or inflammation of the overlying skin and fistulae formation. Radiological investigations are inconclusive and mimic carcinoma, which is the main differential diagnosis at the clinical stage [5]. These investigations may include ultrasound (U/S), mammogram, and magnetic resonance imaging (MRI) [1-3,5,6]. Only positive swab culture and histopathological examination could confirm the diagnosis [1-3]. The treatment choices for GM are dependent on the underlying etiology and may include antibiotics, abscess drainage, wide surgical resection, and even mastectomy [1-3]. In 1991, Hafnia alvei was considered enteropathogenic for the first time [7]. H. alvei mastitis is reported in milking cattle and cows, but no reports were found in humans [8].

## Case presentation

A 41-year-old married lady with three children presented to the clinic six months after her COVID-19 booster dose. She complained of recurrent left breast painful swelling with no associated fever of three years duration. The condition used to settle down by itself or after a course of penicillin antibiotics. Two weeks back, a new painful left breast swelling appeared, associated with fever. The swelling burst two days back and started pouring yellowish discharge. The patient speculates the start of her condition to the booster dose of COVID-19 vaccination, as her breast condition started immediately after. The patient received the COVID-19 Vaccination (Pfizer) first dose on April 2, 2021, the second on April 23, 2021, and the booster dose on August 10, 2021. She had a past surgical history of three lower-segment cesarean sections. She also had a positive family history of breast cancer in two of her aunts. 

**Figure 1:**
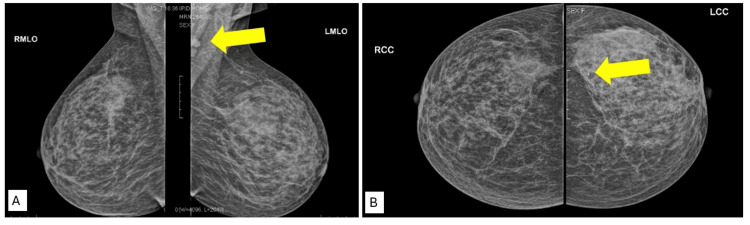
Breast mammogram: left breast benign calcification with duct ectasia and left axillary lymphadenopathy. (A) Arrow points to left axillary lymphadenopathy; (B) arrow points to benign calcifications.

**Figure 2:**
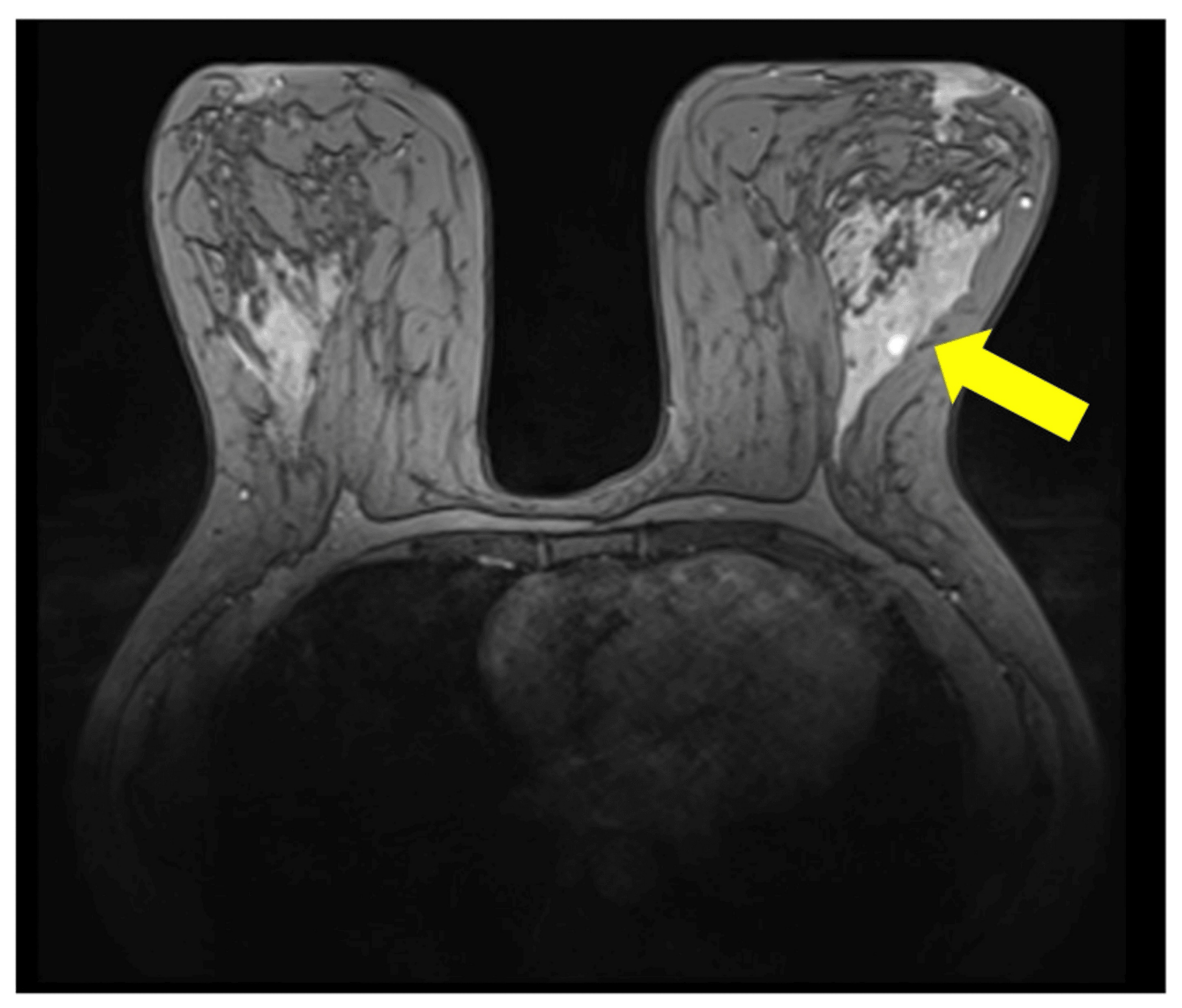
Breast MRI: left breast ectasia, with inflammatory infiltration (mastitis, arrow). MRI, magnetic resonance imaging

She was investigated thoroughly in other hospitals by mammogram, U/S, and MRI breast (Figures 1A-1B, 2) The mammogram showed left breast benign microcalcifications in the upper outer quadrant, duct ectasia, periductal mastitis, and left axillary lymphadenopathy. Breast MRI showed left breast duct ectasia, with inflammatory infiltration suggesting nonspecific mastitis. U/S findings showed only duct ectasia. On her first presentation, three years back, a U/S guided core biopsy was taken and showed acute on top of chronic mastitis.

Her clinical examination revealed a mass in the left breast at the lower outer quadrant around 4 o’clock, 5 x 3 cm in size, with skin sinus pouring pus. Manual evacuation of the abscess through the skin sinus was performed under local anesthesia. A large amount of pus was drained. The pus was sent for culture for aerobes and anaerobes, Ziehl Neelsen stain, culture and polymerase chain reaction (PCR) for acid-fast bacilli, and fungal culture. She was started on Ampicillin and clavulanic acid, 1 g every 12 hours.

The first culture (within 72 hours) grew Streptococcus pyogenes, which was sensitive to the current treatment, yet the patient was not improving clinically. One week later, another rare bacterium, H. alvei bacteria, was cultured from the same sample. It was sensitive to Ciprofloxacin. All other investigations were negative.

**Figure 3:**
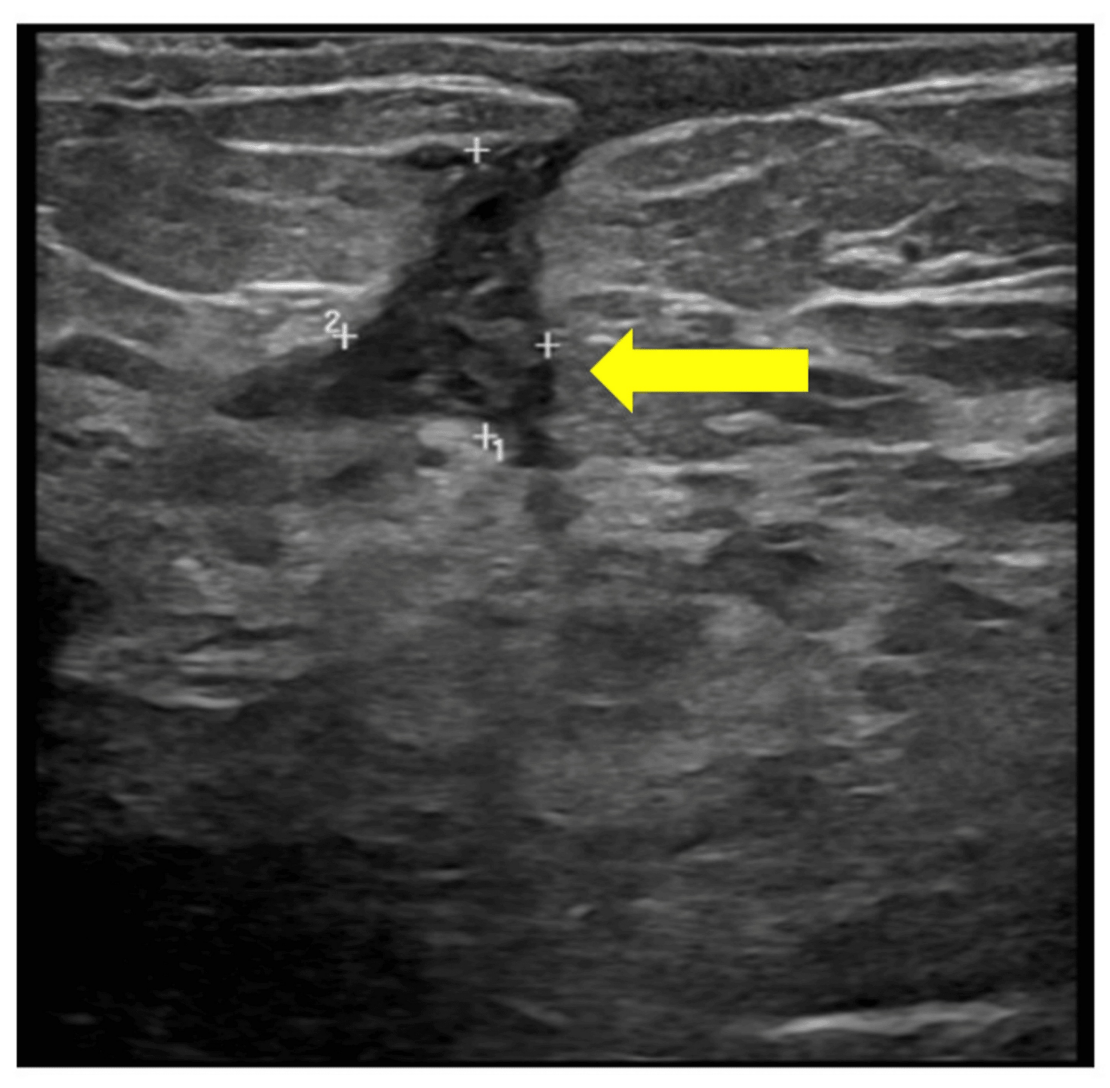
Bilateral breast ultrasound: diffuse edema in the left breast with thickened sinus-like formation (arrow).

Her latest U/S showed diffuse edema in the left breast with periareolar collection and abscess formation. It also showed that the tracts communicated mainly in the retroareolar area. These tracts extended toward the skin with a thickened sinus-like formation (Figure 3).

**Figure 4:**
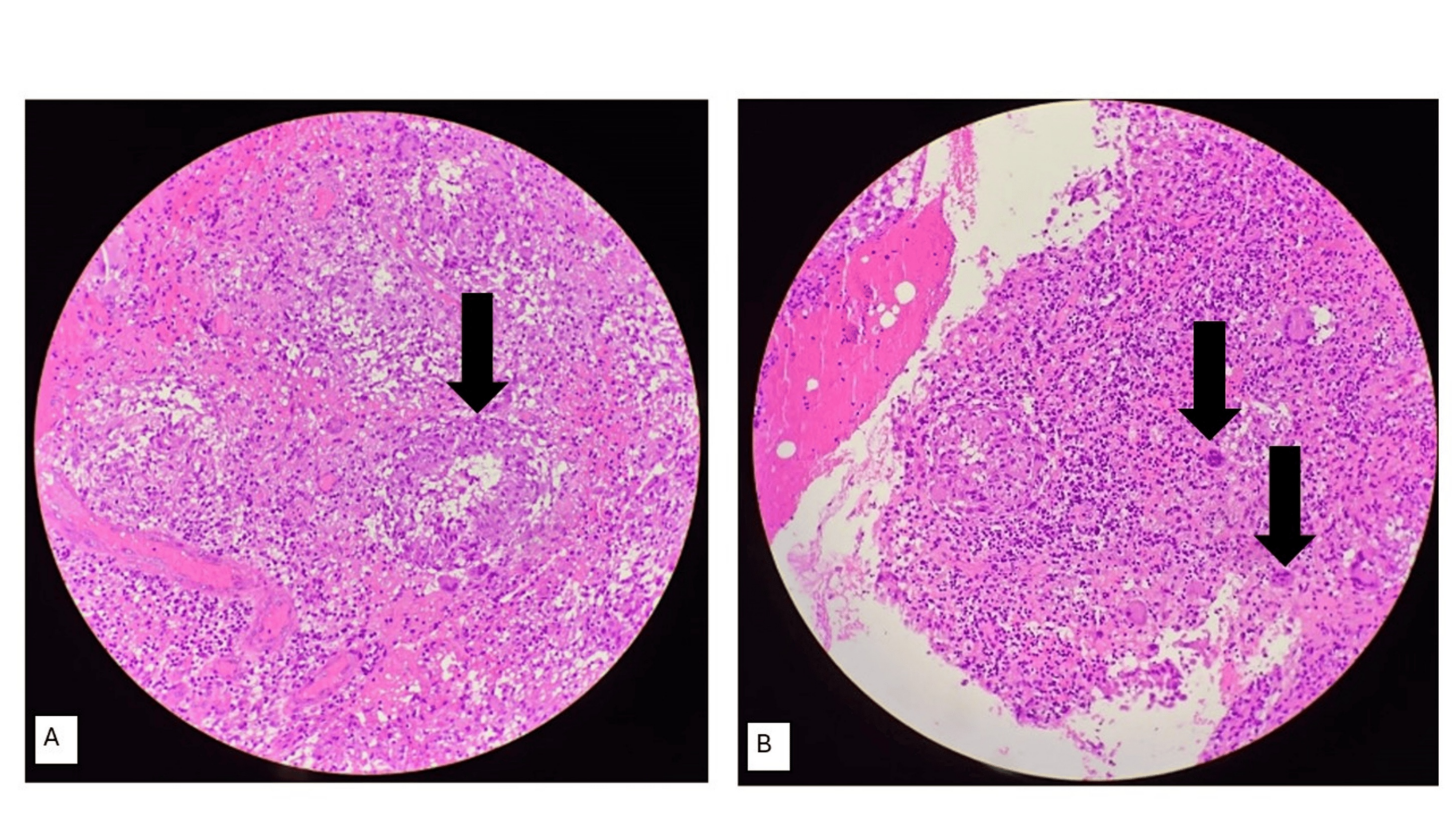
Histopathology of the left breast incisional biopsy showing a few non-caseating epithelioid cell granulomas with surrounding foreign body-type giant cells on a chronic inflammatory background, consistent with granulomatous mastitis. (A) Arrow points to granuloma; (B) arrows point to epithelioid cells.

During treatment, re-collection was not improved in other quadrants, mainly central and upper inner quadrants. Incision and drainage of the breast abscess and incisional biopsy were performed. The biopsy confirmed the diagnosis of non-caseating GM (Figures 4A-4B).

She was started on Ciprofloxacin with regular dressing of the surgical wound and regular follow-up breast U/S. She required multiple sessions of aspiration under U/S guidance. The last cultures were negative, and collections were drying up. She has been on regular follow-up for the previous 10 months. The breast U/S did not show a complete regression of the tracts, but there were no clinical signs of acute relapse. 

## Discussion

GM is defined as an inflammatory breast disease, mostly affecting females of reproductive age [1-3,8,9]. GM can have an unknown etiology. The inflammation could be secondary to trauma, metabolic, hormonal imbalance, autoimmunity, or infections. The infections can be bacterial, mycobacterial, or rarely fungal [1-3]. If it shows caseous necrosis, it is most probably tuberculous GM. This kind of mastitis is common in India and Pakistan [3]. The major etiological hypotheses are ethnicity and autoimmunity [1-3,10,11]. This local inflammatory response becomes chronic, leading to sinus formation and breast disfigurement [1-3].

GM was first diagnosed in 1972 by Kessler and Wolloch as a rare benign breast disease [2]. It is mostly diagnosed in non-Caucasian ethnic groups, with an incidence of 2.4/100,000 women worldwide [2,11]. It is usually unilateral and often appears in the central, retro areolar area but can affect all quadrants of the breast [3,11]. The disease is diagnosed by histopathological examination of breast tissue biopsy. Wolfrum et al. stated that the histopathological report of GM should include granulomas, multinucleated giant cells, epithelioid histiocytes, and plasma cells. In this disease, imaging is inconclusive and can mimic inflammatory carcinoma [4,9,10].

Mammography mostly shows an asymmetrical density, while breast U/S shows frequently hypoechoic mass with tubular tracts pointing towards the skin [5,6,9]. The treatment options are variable due to their unknown etiology. It consists of antibiotics, oral steroids, and surgery [2,5,9,10]. The role of newer options like immunotherapy is yet to be ascertained. It is essential to implement a specific registry for this rare disease to collect more information and put a protocol for diagnosis and management [9,10,12].

Hafnia alvei is a Gram-negative, motile rod belonging to the Enterobacteriaceae family. It is approximately 1 µm in diameter and 2-5 µm in length [4]. H. alvei colonies are sometimes mistaken with Salmonella colonies because both look alike. We can easily differentiate between the two, as Salmonella is Voges-Proskauer (VP) test negative, while almost all H. alvei strains are VP positive at 22 °C [9,12]. H. alvei is an anaerobic bacterium that grows optimally between 30 and 37 °C [4,12,13]. This bacterium habitat is the intestinal tract of animals, particularly mammals. It is cultured in water, milk and dairy products, honey, meat, freshwater fish, soil, and sewage [4,14].

H. alvei has been associated with outbreaks in the poultry industry [13-15]. It has also been reported in horses, fish, snails, birds, insects, and bees; however, more data are needed to understand its significance [13,14].

Asymptomatic carriers are humans and fish and can have H. alvei in their non-phagocytic cells [4,16]. H. alvei in humans is not pathogenic and rarely cultured. It is mainly found in the gastrointestinal tract and is considered an opportunistic bacteria, causing infections in immunocompromised patients [14,17-19]. Although generally extraintestinal, H. alvei infection is very rare; it has been reported to cause gastroenteritis, pneumonia, meningitis, urinary tract infection, bacteremia, nosocomial wound infection, abscesses, septicemia, cholecystitis, endophthalmitis, and arthritis [14,17-19]. 

A descriptive retrospective study was carried out in 2014-2015 on gastroenteritis patients with no associated underlying disease and grew H. alvei on stool culture. Diarrhea was in 57.7% of the cases, and only 43.2% needed treatment. All strains were sensitive to Ciprofloxacin [16].

Studies on children from Bangladesh and Spain showed a high percentage presenting with acute gastroenteritis and diarrhea due to H. alvei. A Finnish study also reported that 16% of tourists returning from Morocco with diarrhea cultured H. alvei in their stools [20].

Kim et al. from South Korea published a case of biliary sepsis caused by H. alvei in a 42-year-old immunocompromised woman known to have metastatic cholangiocarcinoma. She was admitted with sepsis. H. alvei was isolated from bile and blood. The bacteria were sensitive only to piperacillin/tazobactam and ciprofloxacin. The patient's condition worsened, and she only responded to Meropenem [18].

Few studies looked into the systemic involvement of H. alvei. One case of H. alvei urosepsis was reported in an elderly male with a history of long-term catheterization [21]. Another case involved an 11-year-old girl with aortic valve disease and hemolytic-uremic syndrome [22]. Additionally, a case of urinary tract infection was reported in an average child [23].

A one-year-old girl with meningitis and a 20-day-old boy with necrotizing H. alvei enterocolitis and septicemia have also been reported [24]. H. alvei was identified as the causative organism of endogenous endophthalmitis in a patient receiving steroids [25]. H alvei was cultured from a gluteal abscess in a healthy man [26]. There are no recent statistics on the percentage of H. alvei isolation in clinical samples [22].

Treatment of H. alvei infection depends on culture and antibiotic sensitivity testing. Severe cases, usually respond to Imipenem or a third-generation cephalosporin in addition to an aminoglycoside [22].

Méndez et al. reported a case of a 48-year-old previously healthy patient who had a COVID-19 infection admitted to the hospital with type 1 respiratory failure. This patient was on mechanical ventilation in the intensive care unit; five days later, he developed a bacterial co-infection, namely, pneumonia by H. alvei [17].

Opportunistic infections were reported in COVID-19 patients, such as Aspergillus spp., Candida spp., Cryptococcus neoformans, Pneumocystis jiroveci (carinii), Cytomegalovirus, Herpes simplex virus, Mycobacterium tuberculosis, and Toxoplasma gondii. H. alvei were not reported in any case with COVID-19 infection. However, this bacterium can be responsible for severe infections in neonates and adults, especially in hospitalized patients [17].

Two cases of pulmonary infection due to H. alvei were also published. Both patients had underlying comorbidities and were immunosuppressed. The first patient had a history of severe chronic obstructive airway disease, and the second patient had Crohn’s disease [19].

Jung et al. reported a case of spontaneous bacterial peritonitis caused by H. alvei in an elderly male patient with liver cirrhosis secondary to hepatitis C and hepatocellular carcinoma. According to the antimicrobial susceptibility results, H. alvei was isolated from ascetic fluid and responded only to Imipenem and ciprofloxacin [27].

Chronic mastitis in cows was mainly attributed to infection with environmental pathogens that enter the nipple through infected towels, including dirt, water, contaminated milking machines, and poor hygiene practices [8]. Bacillus cereus is usually isolated from cows and can occasionally cause severe, necrotizing mastitis. Candida species, H. alvei, Nocardia spp., Prototheca spp., Serratia marcensens, Pseudomonas aeruginosa, coagulase-negative Staphylococci, and Trueperella pyogenes are causes of chronic mastitis in cows that may be severe and untreatable, leading to significant loss in the milk production industry [4,7,8,13,14].

In our case, H. alvei was only cultured in the patient's last presentation, although the condition of chronic mastitis was diagnosed three years back. H. alvei is an opportunistic bacterium considered non-pathogenic in healthy individuals. We observed that the disease started after taking the booster dose of the COVID-19 vaccine, whether related or not. It could be that the patient had low immunity after the vaccination and got infected with Hafnia. Yamamoto claimed that COVID-19 vaccination is a major risk factor for infections in critically ill patients and recommended discontinuing any booster dose regimen [28]. Backman showed that the mRNA vaccines had a similar risk of triggering autoantibody development. They produce inflammation and more seriously autoantibodies that rarely flare existing autoimmune diseases after vaccination. This may be explained by the phenomenon known as molecular mimicry [29].

To our knowledge, our case is considered the first chronic GM secondary to H. alvei in humans. More studies should be performed to find the source of the bacterium, whether it was food-related or co-infection. 

## Conclusions

We present the first reported case of H. alvei GM in humans. The clinical and radiological aspects were nonspecific and variable. The microbiological identification of the bacteria remains the main element of diagnosis. Treatment of H. alvei infections is guided by antimicrobial susceptibility testing and incision and drainage if associated with abscess. More studies and data about Hafnia and its uprising virulence are needed, such unique cases should be immediately reported to the public health surveillance of the country to monitor for any patterns.

## References

[1] Oze KR, Yehouenou Tessi RT, Mendes P, Allali N, Chat L, El Haddad S (2022). Granulomatous mastitis: a case report. SAGE Open Med Case Rep.

[2] Yukawa M, Watatani M, Isono S (2015). Management of granulomatous mastitis: a series of 13 patients who were evaluated for treatment without corticosteroids. Int Surg.

[3] Bakaris S, Yuksel M, Cιragil P, Guven MA, Ezberci F, Bulbuloglu E (2006). Granulomatous mastitis including breast tuberculosis and idiopathic lobular granulomatous mastitis. Can J Surg.

[4] Frank HJF (2022). Hafnia alvei Is a Gram-Negative, Facultatively Anaerobic, Rod-Shaped Bacterium of the Family Enterobacteriaceae. Encyclopedia of Dairy Sciences, 3rd edition.

[5] Kessler E, Wolloch Y (1972). Granulomatous mastitis: a lesion clinically simulating carcinoma. Am J Clin Pathol.

[6] Lepori D (2015). Inflammatory breast disease: the radiologist's role. Diagn Interv Imaging.

[7] Albert MJ, Alam K, Islam M (1991). Hafnia alvei, a probable cause of diarrhea in humans. Infect Immun.

[8] (2024). SVS Laboratories. Lesser Known Mastitis Pathogens. https://www.svslabs.nz/svs-laboratories-home-sevices-34/.

[9] Gautier N, Lalonde L, Tran-Thanh D (2013). Chronic granulomatous mastitis: imaging, pathology and management. Eur J Radiol.

[10] Wolfrum A, Kümmel S, Theuerkauf I, Pelz E, Reinisch M (2018). Granulomatous mastitis: a therapeutic and diagnostic challenge. Breast Care (Basel).

[11] Altintoprak F, Kivilcim T, Ozkan OV (2014). Aetiology of idiopathic granulomatous mastitis. World J Clin Cases.

[12] Günthard H, Pennekamp A (1996). Clinical significance of extraintestinal Hafnia alvei isolates from 61 patients and review of the literature. Clin Infect Dis.

[13] Padilla D, Acosta F, Ramos-Vivas J, Grasso V, Bravo J, El Aamri F, Real F (2015). The pathogen Hafnia alvei in veterinary medicine: a review. J Appl Anim Res.

[14] Smith JL, Smith J (2014). Hafnia, The Genus. Encyclopedia of Food Microbiology.

[15] Casagrande Proietti P, Passamonti F, Pia Franciosini M, Asdrubali G (2004). Hafnia alvei infection in pullets in Italy. Avian Pathol.

[16] Laupland KB, Church DL, Ross T, Pitout JD (2006). Population-based laboratory surveillance of Hafnia alvei isolates in a large Canadian health region. Ann Clin Microbiol Antimicrob.

[17] Méndez L, Ferreira J, Caneiras C (2021). Hafnia alvei pneumonia: a rare cause of infection in a patient with COVID-19. Microorganisms.

[18] Kim MK, Park JS, Ma DW, Yun GY, Lim JY, Cho JY (2012). A case of biliary sepsis caused by Hafnia alvei in a patient with cholangiocarcinoma. Korean J Med.

[19] Begbey A, Guppy JH, Mohan C, Webster S (2020). Hafnia alvei pneumonia: a rare cause of infection in the multimorbid or immunocompromised. BMJ Case Rep.

[20] Ridell J, Siitonen A, Paulin L, Mattila L, Korkeala H, Albert MJ (1994). Hafnia alvei in stool specimens from patients with diarrhea and healthy controls. J Clin Microbiol.

[21] Yarlagadda K, Shrimanker I, Nookala VK (2019). Catheter-associated Hafnia alvei-induced urosepsis. Cureus.

[22] Crandall C, Abbott SL, Zhao YQ, Probert W, Janda JM (2006). Isolation of toxigenic Hafnia alvei from a probable case of hemolytic uremic syndrome. Infection.

[23] Alaygut D, Bayram A, Soyaltin E (2020). Urinary tract infection caused by Hafnia alvei in a healthy child. Turk J Nephrol.

[24] Mojtabaee A, Siadati A (1978). Enterobacter hafnia meningitis. J Pediatr.

[25] Caravalho J Jr, McMillan VM, Ellis RB, Betancourt A (1990). Endogenous endophthalmitis due to Salmonella arizonae and Hafnia alvei. South Med J.

[26] Agustin ET, Cunha BA (1995). Buttock abscess due to Hafnia alvei. Clin Infect Dis.

[27] Jung SK, Lee JS, Kim KA, Kim YD, Jwa YJ, Kim NK, Kwak YG (2010). Spontaneous bacterial peritonitis caused by Hafnia alvei in a patient with liver cirrhosis. Infect Chemother.

[28] Yamamoto K (2022). Adverse effects of COVID-19 vaccines and measures to prevent them. Virol J.

[29] (2024). Vaccination Has a Lower Risk of Autoantibody Development Than COVID Natural Immunity. https://medicine.yale.edu/news-article/vaccination-has-a-lower-risk-of-autoantibody-development-than-natural-immunity/.

